# Multiplex CRISPR-Cas9 Gene-Editing Can Deliver Potato Cultivars with Reduced Browning and Acrylamide

**DOI:** 10.3390/plants12020379

**Published:** 2023-01-13

**Authors:** Diem Nguyen Phuoc Ly, Sadia Iqbal, John Fosu-Nyarko, Stephen Milroy, Michael G. K. Jones

**Affiliations:** 1 Crop Biotechnology Research Group, School of Agricultural Sciences, College of Environmental and Life Sciences, Murdoch University, Perth, WA 6150, Australia; 2 State Agricultural Biotechnology Centre, Centre for Crop and Food Innovation, Food Futures Institute, Murdoch University, Perth, WA 6150, Australia; 3 Potato Research Western Australia, Murdoch University, Perth, WA 6150, Australia

**Keywords:** CRISPR-Cas9, gene-editing, potato, reducing sugars, acrylamide formation, vacuolar invertase, asparagine synthetase, potato crisps, Atlantic, Desiree

## Abstract

Storing potato tubers at cold temperatures, either for transport or continuity of supply, is associated with the conversion of sucrose to reducing sugars. When cold-stored cut tubers are processed at high temperatures, with endogenous asparagine, acrylamide is formed. Acrylamide is classified as a carcinogen. Potato processors prefer cultivars which accumulate fewer reducing sugars and thus less acrylamide on processing, and suitable processing cultivars may not be available. We used CRISPR-Cas9 to disrupt the genes encoding vacuolar invertase (*VInv*) and asparagine synthetase 1 (*AS1*) of cultivars Atlantic and Desiree to reduce the accumulation of reducing sugars and the production of asparagine after cold storage. Three of the four guide RNAs employed induced mutation frequencies of 17–98%, which resulted in deletions, insertions and substitutions at the targeted gene sites. Eight of ten edited events had mutations in at least one allele of both genes; for two, only the *VInv* was edited. No wild-type allele was detected in both genes of events DSpco7, DSpFN4 and DSpco12, suggesting full allelic mutations. Tubers of two Atlantic and two Desiree events had reduced fructose and glucose concentrations after cold storage. Crisps from these and four other Desiree events were lighter in colour and included those with 85% less acrylamide. These results demonstrate that multiplex CRISPR-Cas9 technology can generate improved potato cultivars for healthier processed potato products.

## 1. Introduction

Cultivated potato (*Solanum tuberosum* L.) is widely grown and, based on global annual production, is the third most important staple food crop after rice and wheat [1]. Potatoes are mainly grown for food, with 80–85% processed into frozen products in Europe and the USA [2]. Global production of processed potato products in more than 70 countries increased from 7.4 million tonnes in 2015 to 8.9 million in 2019 [3]. These products include French fries, chips and crisps, which are usually processed at high temperatures from potato tubers after cold storage.

During cold storage, potato tubers undergo cold-induced sweetening (CIS), a process in which the stored sucrose is converted to glucose and fructose by plant invertases [4]. During high-temperature processing of cold-stored tubers, such as frying, the stored hexose sugars react with free amino acids, such as asparagine, through the Maillard reaction resulting in browning of the potato product. The reaction also generally creates organic compounds, which contribute to the aroma and sweet taste of such processed foods [5]. However, the Maillard reaction can also generate acrylamide, a potential carcinogen and neurotoxin, and various simple carbon compounds associated with the bitter taste and darker colour during the high-temperature processing of potato products [6]. The level of browning and acrylamide in such processed potato products can be reduced if hexose sugars and non-essential amino acids accumulated in cold-stored tubers are low [7]. However, because the storage of potato tubers at low temperatures is needed for continuous supply and production of processed potato products, cultivars with a reduced potential to accumulate reducing sugars are desired by potato processors [8,9]. This is because management strategies to reduce discolouration and acrylamide formation during high-temperature processing are not always practical [10]. Despite over 4780 accessions of potatoes held at the International Potato Centre [11], and other germplasm collections, which provide diverse resources for breeding cultivars with better high-temperature-processing properties, conventional breeding has yet to deliver commercial cultivars with all the necessary traits for high-temperature processing. For example, cv. Atlantic, a popular chipping potato variety in North America, still accumulates reducing sugars during long-term storage [12,13]. It is, therefore, essential that new methods for developing good quality potato cultivars for high-temperature processing are sought. 

Incorporating traits in potatoes so heat-processed products have low acrylamide-forming potential and reduced browning has been attempted using molecular tools. These have involved strategies to inhibit the activity of the vacuolar invertase (VINV), a key enzyme that hydrolyses sucrose to glucose and fructose in cold-stored tubers, and the two isoforms of asparagine synthetase (AS1 and AS2), the enzymes responsible for the catalysis of asparagine from aspartate and glutamine [14,15,16]. For example, RNA interference (RNAi) of the *AS1* gene has been reported to reduce the production of asparagine, with some potato lines also having up to 70% less acrylamide than wild-type products, whereas RNAi of the *AS2* gene does not appear to decrease asparagine synthetase level and can negatively impact plant growth [15,17]. Moreover, switching off the *VInv* or *AS1* gene alone does not always guarantee the desirable reduction in acrylamide formation [15]. Simultaneously reducing or eliminating the expression of the *VInv* gene or the activity of the encoded enzyme and the *AS1* gene will likely reduce the accumulation of hexose sugars and possibly slow down the rate of asparagine synthesis leading to the reduced acrylamide-forming potential in cold-stored tubers. This was demonstrated using RNAi to silence the *VInv, AS1* and *AS2* genes, resulting in only one-fifteenth of the acrylamide content in controls in the best RNAi lines [18]. However, knocking out the *AS2* gene resulted in some RNAi lines being stunted and chlorotic during field trials, confirming earlier observations that the *AS2* may not be a good candidate for genetic manipulation aimed at reducing just the levels of asparagine [18]. RNAi technology is often associated with incomplete silencing of target genes in various organisms [17,18,19,20,21]. As such, gene editing tools that can permanently and completely reduce the activity of target enzymes by modifying genes are a better option than RNAi. An example is the use of the transcription activator-like effector nucleases (TALENs) to knock out the *VInv* in the commercial potato cultivar Ranger Russet, resulting in some edited lines having undetectable levels of reducing sugars and the processed chips being light-coloured and containing reduced levels of acrylamide [22]. In all of the above-reported cases, the lines created are transgenic, as they have foreign DNA, although some of the TALENs-edited lines were suggested to contain no TALEN DNA in the genome [22]. It is now possible to use another gene editing tool, the Clustered Regularly Interspaced Short Palindromic Repeat (CRISPR) system, to introduce mutations that will permanently eliminate the expression of genes, as with TALENs; but, unlike TALENs, it is simpler, more versatile, more efficient and can achieve similar outcomes with or without foreign DNA [23]. 

The CRISPR editing system is a natural antiviral system used by bacteria and has been modified as a tool to edit plant genes [24,25]. With this approach, a DNA vector, encoding a plant codon-optimised CRISPR endonuclease (e.g., the Cas9 protein) and a guide RNA (gRNA) complex, is expressed in plant cells where a ribonucleoprotein (RNP) complex formed between the endonuclease and gRNA is directed to cleave target DNA. The gRNA complex comprises a 17–20 bp single guide RNA (sgRNA), complementary to the target DNA sequence, and fused to a gRNA scaffold. The sgRNA replaces the spacer sequences called CRISPR RNA (crRNA), and the gRNA scaffold mimics the trans-activating CRISPR RNA (tracrRNA) found naturally in the bacteria antiviral system. The sgRNA is designed based on knowledge of the protospacer adjacent motif (PAM), the recognition site of a CRISPR endonuclease. Typically, in the RNP complex, the tracrRNA binds to the CRISPR endonuclease, while the sgRNA guides the complex to the target DNA, which cuts at specific sites adjacent to the PAM. For the CRISPR-Cas9 system, which employs the CRISPR-associated protein 9 as the CRISPR endonuclease, the sgRNA directs the RNP to cleave both DNA strands at three nucleotides upstream of the PAM, which is usually NGG. Endogenous DNA repair processes at the cleavage site by non-homologous end-joining (NHEJ) can result in deletions, insertions or substitutions at that site. When this repair results in a frameshift or nonsense mutation, the gene product is impaired or inactivated. Since the first report a decade ago, the CRISPR-Cas9 system has been used to successfully edit genes of many plants, including potatoes [26]. A recent advance of the technology is the introduction of mRNA of the Cas9 and gRNA complex or an externally assembled RNP to edit plant genes without introducing foreign DNA, making it possible to obtain non-GM (Genetically Modified)-edited plants. These applications make the CRISPR-Cas system a valuable alternative to generating gene-edited transgenic plants, especially in countries where transgenic plants are still regulated or are not favoured by consumers. 

The potato genome is amenable to CRISPR-Cas9 editing, and the technology has been used to introduce herbicide and pathogen resistance [27,28,29], remove self-incompatibility [30,31], and alter tuber chemical composition and nutritional value [32,33,34]. To increase the efficiency of the CRISPR-Cas9 system, more than one gRNA can be introduced to edit alleles of a gene or multiple genes at similar or different chromosomal locations in a genome, for example, in the tetraploid potato. Successful multiplex gene editing is necessary because a change in polygenic traits may require modifications in more than one gene or all alleles of a gene or genes in a biological or metabolic pathway before a meaningful physiological change is achieved [32,35]. Multiplex editing can facilitate the development of multiple traits or improve existing ones in established cultivars. In several recent studies, multiplex gene editing has also been applied to edit target genes in potatoes [32,33,36]. 

This study is the first report on the application of the CRISPR editing system, specifically multiplex CRISPR-Cas9 editing, to demonstrate that the tool can rapidly generate cultivars with improved quality traits for high-temperature processing of potato products. We used the most common transgenic method involving DNA vectors encoding the Cas9 protein and multiple guide RNAs to mutate the *VInv* and *AS1* genes in two potato cultivars as a proof-of-concept. In addition, we explored the non-transgenic method of inducing mutations, which involved the delivery of RNPs assembled outside of plant cells. We demonstrated that mutations in some events resulted in reduced browning and low acrylamide in crisps made from cold-stored tubers. While the transgenic gene-edited events are valuable, the RNP strategy demonstrates that it is possible to generate edited potato varieties with desirable commercial traits without introduced DNA, and, in jurisdictions which do not regulate them as genetically modified, it will be cheaper to bring them to market, and processed products from them may be readily acceptable to consumers. 

## 2. Results

### 2.1. Specific Guide RNAs Cleaved the VInv and AS1 Genes 

**Figure 1:**
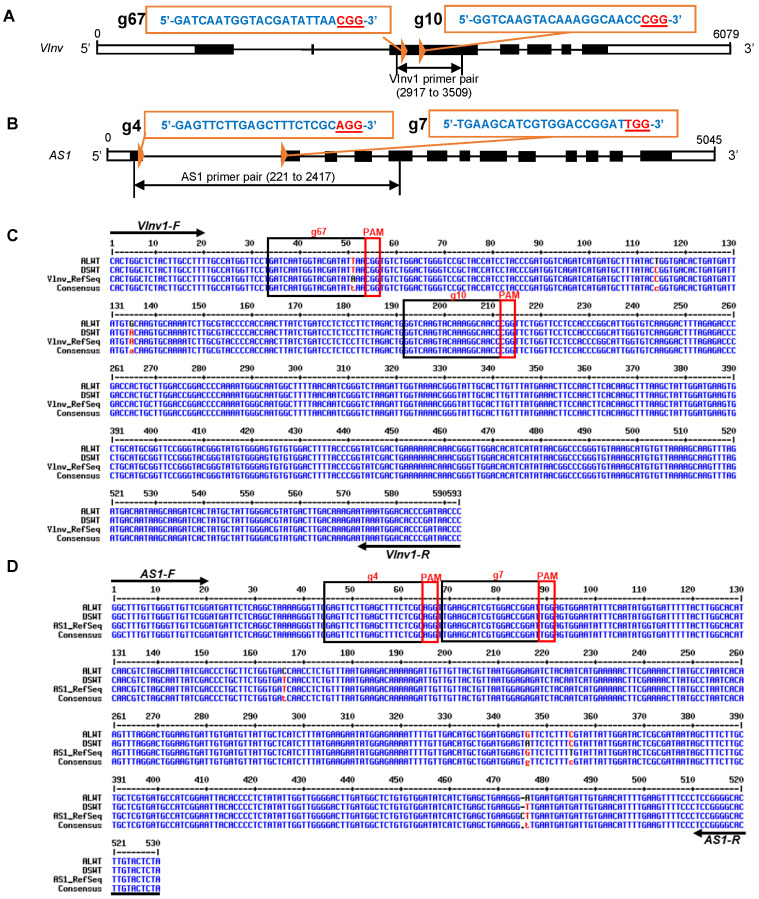
Gene organisation and selected sgRNA sequences of the vacuolar invertase (VINV) and the asparagine synthetase 1 (AS1) genes of potato. (**A**) The structure of the *AS1* gene showing sgRNA sequences and primers flanking exons 1 to 5. (**B**) The structure of the *VInv* gene showing sgRNA sequences and primers used to amplify a fragment of exon 3. For both structures, the white boxes represent the untranslated region, the black boxes represent exons and the black lines represent introns. Sequences of the target sites are shown in blue, and the protospacer adjacent motifs (PAM) are in red, underlined. (**C**) Alignment of consensus sequences of clones of the amplicon of the *VInv* gene of Atlantic and Desiree cultivars with the NCBI reference sequence showing the sgRNA sequences (black box), associated PAMs (red box) and primers (arrows with names on top) used for PCRs of the amplicon. (**D**) Alignment of consensus sequences of clones of the amplicon of the *AS1* gene of Atlantic and Desiree cultivars with the NCBI reference sequence showing the sgRNA sequences (black box), associated PAMs (red box) and primers (arrows with names on top) used for PCRs of the amplicon.

The complete sequence of the *VInv* gene of potato targeted for editing in this study was 6097 nucleotides (nt) long and had 5′ and 3′ UTRs and seven exons (Figure 1A). The *AS1* target gene was 5045 nt long with 5′ and 3′ UTRs and 13 exons (Figure 1B). The target sites for editing the *AS1* were in the first and second exons, whereas those for *Vlnv* were selected from the third exon (Figure 1A,B). To confirm the selected sites were conserved in all alleles of the genes, a 593-nt region from the third exon of the *VInv* gene and a 530-nt region spanning exons 1 to 5 of the *AS1* gene were amplified from genomic DNA of the potato cultivars Atlantic and Desiree, cloned and analysed to identify polymorphism(s) (Figure 1C,D). Because the intervening introns between exons 1 to 5 of the *AS1* gene were large, the clones for this gene were made from cDNA. For each gene, there was a 97% similarity between the clones from both cultivars. Sequences of both genes for cvs. Atlantic and Desiree were 95% identical to the respective reference sequences at the National Centre for Biotechnology Information (NCBI) (Figure 1C,D). The sequences generated from the Atlantic and Desiree wild-types were used as references for identifying mutations in gene-edited lines generated in the project. We chose Atlantic and Desiree for the study because both are popular commercial potato varieties. In addition, Desiree is responsive to tissue culture, whereas Atlantic is a chipping variety and, as such, an excellent variety to gauge how much improvement can be made with the CRISPR technology. 

Two sgRNAs were designed to target the sense strand of each gene. The target sites were selected from conserved sequences between the cultivars Atlantic and Desiree to ensure that the sgRNAs would edit the target genes of both cultivars. The sgRNAs were g67 and g10, 135 nt apart and targeting exon 3 of the *VInv* gene, and g4 and g7 for the *AS1* gene, with target sites 1163 nt apart in exons 1 and 2, respectively (Figure 1). 

**Figure 2:**
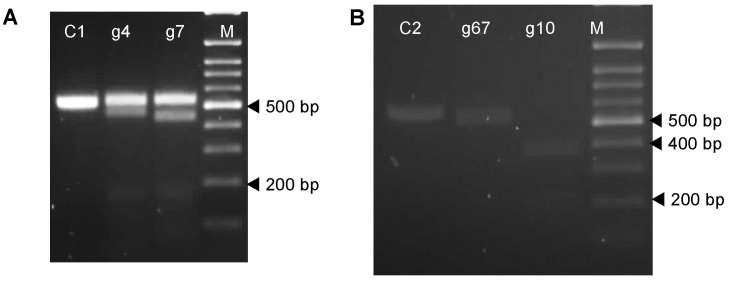
Representative gel images of in vitro Cas9 cleavage assays of the vacuolar invertase (VINV) and the asparagine synthetase 1 (AS1) genes of potato. (**A**) Gel image of untreated and cleaved fragments of amplicons of the AS1 gene. Lane C1: DNA (530 bp amplicon generated from cDNA); Lane g4: Cas9 + DNA + g4; Lane g7: Cas9 + DNA + g7; Lane M: 100 bp DNA ladder. The expected sizes of cleaved products were 472 bp and 61 bp in lane g4, and 445 bp and 85 bp in lane g7, (**B**) Gel image of untreated and cleaved fragments of amplicons of the *VInv* gene. Lane C2: DNA (593 bp amplicon generated with the vlnv1 primers). Lanes g67: Cas9 + DNA + g67; Lane g10: Cas9 + DNA + g10; Lane M: 100 bp DNA ladder. The expected sizes of cleaved products were 543 bp and 50 bp in lane g67 and 385 bp, and 208 bp in lane g10.

Further comparative analysis of the guide sequences with publicly available genomic and transcriptomic sequences of potatoes indicated none of the four guides had any potential off-target sites in the potato genome. In vitro Cas9 cleavage assays were used to assess the efficiency of the four sgRNAs by incubating the target amplicons of both the *VInv* (593 bp) and *AS1* (530 bp) of the Atlantic and Desiree cultivars with Cas9 and in vitro transcripts of the gRNA complex for each sgRNA, which consisted of the sgRNA linked to a gRNA scaffold. Gel electrophoresis analyses indicated that the amplicons of both genes were cleaved into the expected fragments indicating that each of the RNPs formed with each of the four sgRNAs was functional (Figure 2A,B).

### 2.2. The RNP Complex of Cas9 and Four sgRNAs Induced Mutations at a Frequency of 0.4% 

**Figure 3:**
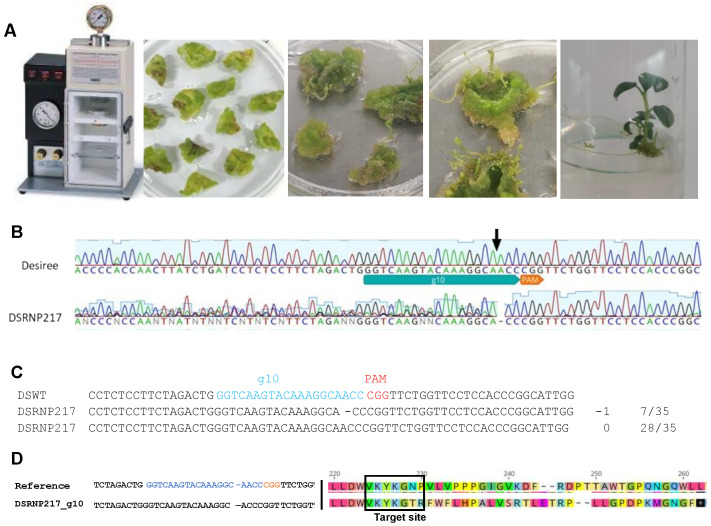
Single guide RNA-directed mutations in *VInv*-edited event DSRNP217. (**A**) Images of the RNP particle bombardment equipment, bombarded leaf discs and development of event DSRNP217. (**B**) Part of the chromatogram of the *VInv* sequence of Desiree wild-type (DSWT) and edited event DSRNP217. The black arrow indicates the mutation site relative to the PAM (protospacer adjacent motif). (**C**) Partial alignment of the *VInv* sequence of Desiree wild-type (DSWT) and edited event DSRNP217 showing a base deletion at the g10 target site and the fraction of clones with the genotype. The sgRNA sequence is shown in blue text, and the PAM is red in the DSWT sequence. (**D**) Translated sequence of a region of the *VInv* gene of the wild-type and the edited event DSRNP217 showing changes in the amino acid at the target cleavage site and the introduction of a premature stop codon (white asterisk in black box).

A total of 294 calli derived from pre-cultured leaf discs (206 explants from Atlantic and 88 from Desiree) were subjected to particle bombardment using a complex of Cas9 and the four sgRNAs. Shoots emerged from the treated explants two weeks after bombardment and were transferred to a fresh shooting medium and then to a rooting medium without a selection agent (Figure 3A). Potential mutations in the generated plants were determined from sequences of three amplicons; the first was 385 bp covering a region containing the g67 and g10 target sites (*VInv* gene), the second, 191 bp covering a region containing the g4 target site (*AS1* gene) and the third amplicon was 407 bp covering a region containing the g7 target site (*AS1* gene). When amplicon sequencing results indicated a mixed template, the amplicon was cloned. For such amplicons, between 18 and 35 clones were sequenced to confirm mutations in alleles. Results from sequences of 343 plantlets (166 Atlantic and 227 Desiree) showed that compared to the Desiree wild-type (DSWT), one Desiree event, DSRNP217, contained a putative mutation (Figure 3B). This represented an editing frequency of 0.4% for the cv. Desiree for the RNP approach. For this event, 20% of the 35 clones sequenced had a one-base deletion at the fourth position upstream of the PAM at the g10 target site (Figure 3C). The translated wild-type VINV protein sequence was 639 amino acids long. The single base deletion resulted in a premature stop codon (PSC) at the 263rd position on the translated VINV protein sequence (Figure 3D). No mutation was detected in sequences of amplicons at any of the other three target sites, implying the *AS1* was not edited.

### 2.3. Agrobacterium Carrying DNA Vectors Encoding Cas9 and Multiple sgRNAs Edited Both the VInv and AS1 Genes 

To maximize the chances of generating potato events with intended mutations, two gene-editing DNA vectors, each encoding a form of plant codon-optimised Cas9 sequence but with different translation and transcription regulatory elements driving the Cas9 and the respective *BlpR* and *nptII* genes as selectable markers, were used for potato transformation via the *Agrobacterium*-mediated transformation method (Supplementary Figure S1). The vectors were generated using the parent gene editing vectors pFGC-pcoCas9 and pFN117-Cas9 and were modified with a sgRNA cassette, ASV1, to form pFGC-pcoCas9-ASVI and pFN117-Cas9-ASVI. The Cas9 coding sequence in the pFN117-Cas9-ASVI is driven by two repeats of the cauliflower mosaic virus (CAMV) 35S promoter fused to a translational enhancer from the tobacco mosaic virus 5′-leader sequence, whereas the Cas9 in the pFGC-Cas9-ASVI vector is driven by 35SPPDK, a hybrid promoter consisting of the CAMV 35S promoter fused to the maize C4PPDK basal promoter. In addition, the Cas9 coding sequence in pFGC-Cas9-ASVI is interrupted with a 189 bp intron; a modified second intron of the potato ST-LS1 gene (Supplementary Figure S1). 

**Figure 4:**
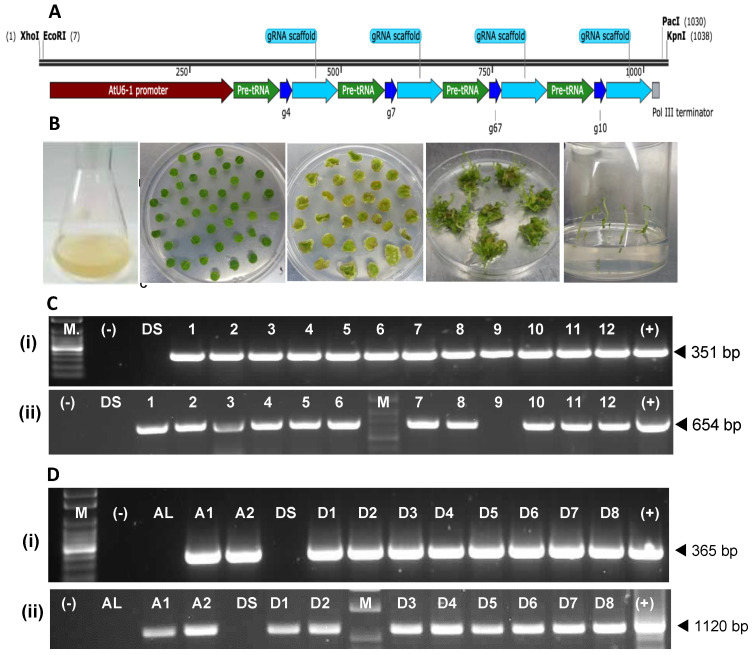
Generation of transgenic events of potato cultivars Atlantic and Desiree. (**A**) The gRNA construct, ASVI, used to modify the gene editing vectors pFGC-pcoCas9 and pFN117-Cas9. (**B**) Modified Agrobacterium culture and stages of development of treated leaf discs through to shoot and root development in selection media. (**C**) Gel images of amplicons of Cas9 (**i**) and the *BlpR* (**ii**) genes from events DSpco1 to Dspco12 (1—12 on gel). Lane M = DNA ladder; lane (—) = no DNA control; lane (+) = Plasmid DNA control; lane DS—Wild-type Desiree (**D**) Gel images of amplicons of Cas9 gene (**i**) and the ASVI construct (**ii**) from events ALpFN1 (A1), ALpFN2 (A2), and DSpFN1 to DSpFN8 (D1 to D8 on gel). Lane M = DNA ladder; lane (—) = no DNA control; lane (+) = Plasmid DNA control; lane DS = Wild-type Desiree; lane AL = Wild-type Atlantic.

The ASVI cassette consisted of a tRNA sequence linked to each sgRNA sequence and a sgRNA scaffold, arranged in tandem and the complex driven by the Arabidopsis U6-1 promoter (Figure 4A). *A. tumefaciens* modified with vector pFGC-Cas9-ASVI was used to treat 156 Atlantic and 294 Desiree leaf discs whereas 925 Atlantic and 403 Desiree leaf discs were treated with *A. tumefaciens* harbouring the pFN117-Cas9-ASVI vector (Figure 4B). From these, 22 putative transgenic events, which survived the antibiotic selection, were generated. Two events for cv. Atlantic, ALpFN1 and AlpFN2 harboured the vector pFN117-Cas9-ASVI. The same vector also yielded eight Desiree events (designated DSpFN1 through DSpFN8). The other 12 Desiree events, Dspco1 through Dspco12, were transformed with pFGC-pcoCas9-ASVI. 

PCRs were used to confirm the transgenic status of the events. Amplification of 351 bp and 365 bp fragments of the Cas9 DNA from the events generated with pFGC-pcoCas9-ASVI and pFN117-Cas9-ASVI, respectively, indicated all 22 events had the Cas9 coding sequence integrated into the genomes of the events (Figure 4C(i),D(i)). Moreover, the amplification of a section of the T-DNAs (including the *BlpR* gene) from genomic DNA indicated 11 of the 12 events generated with the pFGC-pcoCas9-ASVI were transgenic; the exception was event DSpco9, for which the expected 654 bp amplicon was not obtained from the 100 ng of DNA used as template, although the Cas9 gene was amplified from the genome of the event (Figure 4C(ii)). Similarly, a 1120 bp of the T-DNA of vector pFN117-Cas9-ASVI, covering the gRNA cassette, was amplified from DNA of all ten events generated with pFN117-Cas9-ASVI, further confirming their transgenic status (Figure 4D(ii)). 

Mutations in the transgenic plants were assessed using the sequences of amplicons that covered the target cleavage sites of both the *AS1* and *VInv* genes. For amplicons where the chromatograms indicated mixed templates, the amplicons were cloned, and the mutation frequencies were estimated based on the number of clones with observed mutations. Based on the sequence analyses, ten of the 12 DSpco events were edited, and this number included event DSpco9 for which the Cas9 and not the *BlpR* gene was amplified from the genome. No edits were evident for events DSpco1 and DSpco6. Events DSpco5 and DSpco12 had mutations only in the *VInv* gene, whereas the remaining events had mutations in both genes. In addition, events ALpFN1, ALpFN2 and DSpFN1 through to DSpFN8 all had mutations in both target genes. A summary of the events and the status of mutations in the genes are summarized in Table 1. 

**Figure 5:**
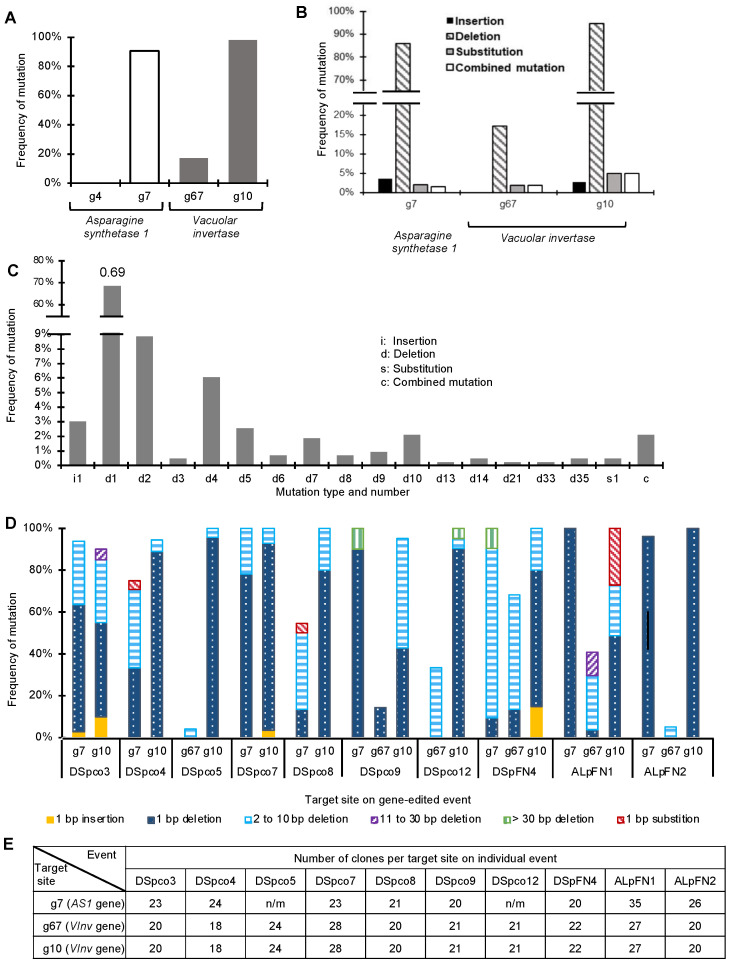
Mutation frequencies and types induced by the four sgRNAs. (**A**) Mutation frequency at sites targeted with the sgRNAs g4, g7, g67, and g10. (**B**) Frequency of detected mutations at three target sites; g7, g67, and g10. (**C**) Type and frequency of the mutations detected in the events. The numbers after the alphabets on the x-axis indicate the number of edited nucleotides, e.g., i1 indicates one base insertion. The frequency of mutation type was calculated based on the number of clones carrying that type of mutation divided by the total clones with mutations. (**D**) Frequency of induced mutation types in ten edited events of Atlantic and Desiree. The frequency of a mutation type at a particular target site was calculated by dividing the number of clones carrying that mutation by the total number of sequenced clones, as shown in (**E**). n/m: no mutation detected.

Gene-editing in ten events, DSpco3, DSpco4, DSpco5, DSpco7, Dspco8, DSpco9, DSpco12, DSpFN4, ALpFN1 and ALpFN2, were analysed in detail; the stage of development of these regenerated events was similar, and this made them good candidates for physiological experimentation. The mutation frequency at each target site was estimated as the number of sequences of clones (of an amplicon) with mutations per the total number of clones sequenced. The highest mutation frequency was recorded for the g10 sgRNA for *VInv* (98%), followed by the g7 sgRNA (91%) for *AS1* and then the g67 sgRNA (17%) on the *VInv* gene (Figure 5A). No mutations were evident at or around the g4 sgRNA site on the *AS1* gene (Figure 5A). The most common mutation type induced by the sgRNAs was deletion, ranging from 17% for the g67 gRNA to 95% associated with the g10 sgRNA (Figure 5B). Insertions, substitutions, and combined mutations were detected at frequencies less than 5% (Figure 5C). The deletions ranged from 1–35 bases, with one base deletion being the most common among the edited sequences (69%, Figure 5C). The insertions and substitutions were limited to one base and occurred at a much lower frequency (3% insertions and 0.5% substitutions, Figure 5D). The absence of wild-type sequences among the clones for a target site indicated that all alleles of the target gene were edited at that site. This was the case for four events (DSpco5, DSpco8, DSpco12, and ALpFN2) at the g10 target site in the *VInv* gene and DSpco9 at the g7 target site in the *AS1* gene. Interestingly, for DSpco7, DSpFN4 and ALpFN1, the sequence analyses indicated that all alleles of the *AS1* and *VInv* genes were fully edited at the g7 and the g10 target sites (Figure 5D,E).

**Figure 6:**
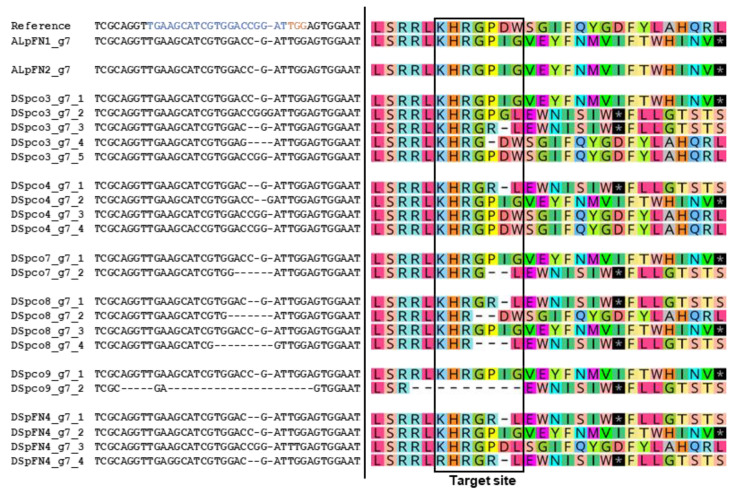
Alignments of partial nucleotide sequences of the *AS1* gene showing mutations at the g7 target site for (clones of) eight edited events and translation of some nucleotide sequences around the g7 sgRNA. Reference = GenBank accession nos.: XM_006343993.2, NW_006238977.1, also the wild-type sequence. The g7 sgRNA sequence is coloured blue, and the protospacer-adjacent motif is in orange. The * with a black background in the protein sequences represents stop codons. Each sequence represents an allele of the gene at the target site.

**Figure 7:**
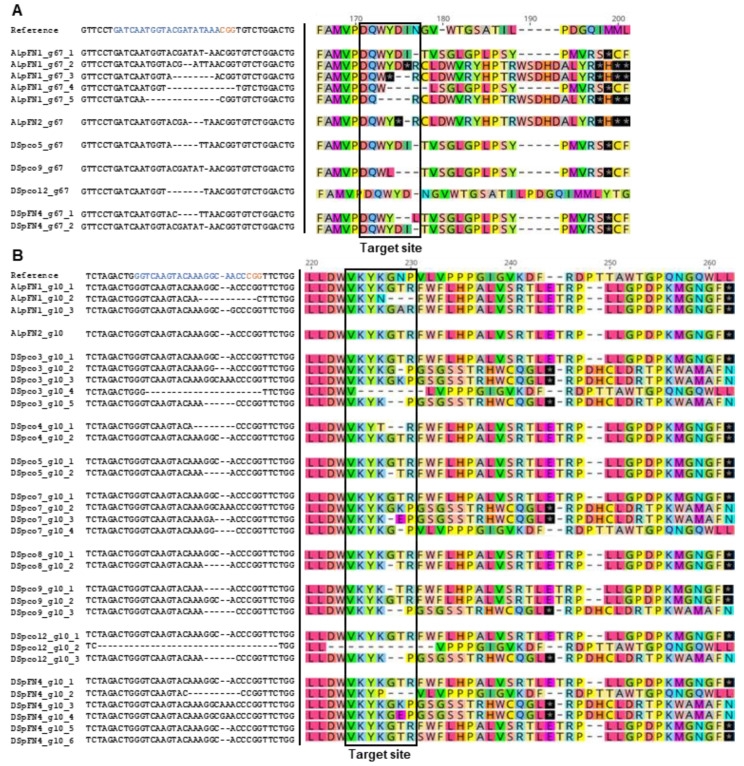
Partial alignment of nucleotide sequences of the *VInv* gene and partial translated sequences of the VINV of edited potato events around the (**A**) g67 target site and (**B**) g10 target site. Reference = GenBank accession no.: HQ110080.1, also the wild-type sequence. The sgRNA sequence is shown in blue, and the protospacer-adjacent motif is in orange. The * with the black background in the protein sequences represents stop codons. Each sequence represents an allele of the gene. The gaps in the wild-type protein sequences were created to accommodate all mutations in the edited events.

In silico protein translation of the mutated sequences revealed PSCs in alleles of some target genes. The wild-type AS1 protein is 590 amino acids long, and in the *AS1*-edited events, the alleles were predicted to have PSCs between positions 34 to 51 of the translated amino acid sequence (Figure 6). The translated AS1 proteins of events ALpFN1, ALpFN2, DSpco7 and DSpco9 had PSCs in all the edited alleles, while the mutations in events DSpco3, DSpco4, DSpco8 and DSpFN4 resulted in changes in amino acids (Figure 6). The g67-induced mutations in event ALpFN1 resulted in PSCs in the translated VINV protein sequence for the mutated alleles close to the target site, for example, at the 174th position (Figure 7A). Except for DSpco3, DSpco7, DSpco12 and DSpFN4, all other events were predicted to have PSCs in the translated amino acid sequences of the *VInv* gene (Figure 7A,B).

### 2.4. Mutations in the VInv Gene Result in Low Levels of Hexose Sugars in Some Cold-Stored Tubers

**Figure 8:**
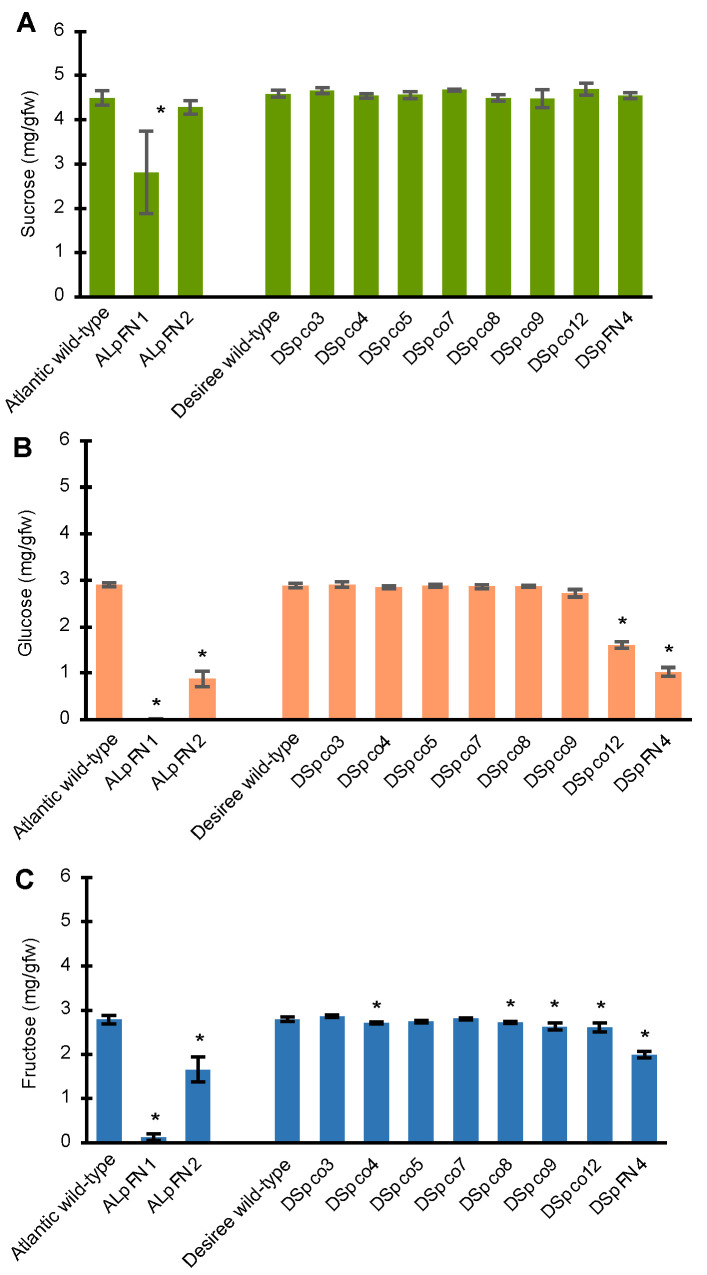
Effect of gene editing of the *VInv* gene on the concentrations of sucrose, glucose and fructose in cold-stored tubers of Atlantic and Desiree events. Mean concentrations of (**A**) sucrose, (**B**) glucose and (**C**) fructose in wild-type and edited events. The bars represent the means and standard deviations of three replicate tubers and three technical measurements for each replicate tuber. The asterisks (*) indicate concentrations significantly (*p* < 0.05) different from those of the wild-type.

Tubers of the two Atlantic double *VInv*/*AS1*-edited events and eight Desiree events (six double *VInv*/*AS1*-edited events and two *VInv*-edited events) were analysed for the effect of mutations on the accumulation of hexose sugars in tubers stored at 4 °C for four months. There was a significant reduction (*p* < 0.05) of 32.4% in sucrose concentration in event ALpFN1 compared to that of wild-type tubers (Figure 8A). The sucrose concentration in tubers of the ALpFN2 event and all the Desiree events was not significantly different from that of the corresponding wild-type tubers (Figure 8A). The glucose and fructose levels in cold-stored tubers of ALpFN1 and ALpFN2 were significantly lower than in tubers of the Atlantic wild-type (*p* < 0.05). In particular, the concentrations of glucose and fructose in tubers of ALpFN1 were 99% and 95% less than in the wild-type (Figure 8B,C). There was significantly more fructose than glucose in tubers of both ALpFN1 and ALpFN2. However, the concentrations of both sugars in the two events were significantly lower than in wild-type tubers, with the levels of glucose, for example, being 0.13 mg/gfw in ALpFN1 and 1.66 mg/gfw in ALpFN2 compared to 2.79 mg/gfw in the wild-type (*p* < 0.05; Figure 8C). 

Two of the eight Desiree events, DSpco12 (*VInv*-edited) and DSpFN4 (*VInv/AS1*-edited), accumulated less glucose and fructose in the cold-stored tubers, with respectively 55.4% and 35.6% less glucose than the wild-type (*p* < 0.05, Figure 8C). However, tubers of three other Desiree events (DSpco4, DSpco8 and DSpco9) contained significantly less fructose than in stored tubers of the wild-type (*p* < 0.05), but not less glucose.

### 2.5. Fried Crisps of VInv-and AS1-Edited Events Had Reduced Browning and Less Acrylamide 

**Figure 9:**
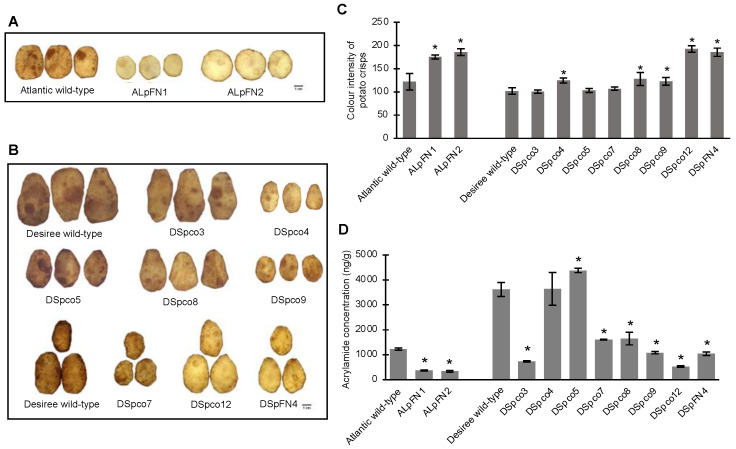
Quality of crisps made from *VInv* and/or *AS1*-edited events of Atlantic and Desiree. Crisps made from (**A**) Two double *VInv*/*AS1*-edited Atlantic events and (**B**) Eight edited events of Desiree (*VInv*-edited: DSpco5 and DSpco12; double *VInv*/*AS1*-edited: DSpco3, DSpco4, DSpco7, DSpco8, DSpco9 and DSpFN4). The two images of the Desiree wild-type were from different batches of the frying process; the similar colour of crisps indicated the consistency of the frying process. (**C**) Quantitative measurement of the colour intensity of the crisps made from the events. The bars represent the means and standard deviations of the colour intensities assigned by the FIJI image processing software, which gives a higher value to the lighter colour. (**D**) Acrylamide content in fried crisps made from the events. Asterisks (*) indicate values significantly different (*p* < 0.05) from that of the wild-type.

The effect of mutations on the quality of crisps made from cold-stored tubers of the gene-edited events was assessed based on the level of browning and the acrylamide concentration in the fried crisps. Replicates of cold-stored tuber slices from the ten edited events described above were deep-fried at 191 °C for 60 s, and the colour intensity was compared to the corresponding wild-type slices in the same frying batch. The crisps made from the two Atlantic events, ALpFN1 and ALpFN2, with significantly lower fructose and glucose levels, were lighter in colour than crisps made from the wild-type tubers (Figure 9A). Similarly, crisps from five Desiree events were lighter in colour (less brown) than the wild-type (Figure 9B). The visual observations were confirmed by comparing the colour intensities of the crisps using values assigned by the FIJI software, which gives higher values to lighter colours [37]. Based on the mean values, the colour intensities of the crisps made from both Atlantic (ALpFN1, ALpFN2) and five of the Desiree (DSpco4, DSpco8, DSpco9, DSpco12 and DSpFN4) events were significantly different from the wild-type (*p* < 0.05, Figure 9C). Crisps of the other three Desiree events, DSpco3, DSpco5 and DSpco7, exhibited similar browning to the crisps made from the wild-type tubers (*p* < 0.05, Figure 9C).

The mean acrylamide concentration in crisps made from tubers of eight of the ten Atlantic and Desiree events was significantly lower than those of the wild-type (*p* < 0.05). The mean concentrations in crisps of the two Atlantic double *VInv*/*AS1*-edited events, ALpFN1 and ALpFN2, were respectively 72.8% (365.4 ng/g) and 70.1% (332.9 ng/g) lower than in the wild-type (1222 ng/g, Figure 9D). Similarly, six Desiree events (DSpco3, DSpco7, DSpco8, DSpco9, DSpco12 and DSpFN4) produced crisps with significantly (*p* < 0.05) lower acrylamide concentrations (Figure 9D) compared to the wild-type (3614 ng/g) and ranged from 54.4% less in DSpco8 (1647 ng/g) to 85.3% less in DSpco12 (529.9 ng/g), the event with the lowest acrylamide concentration among the Desiree crisps. Interestingly, DSpco12 and DSpco5, each of which had only the *VInv* edited, had significantly different acrylamide levels, the former with a much lower content and the latter with a much higher content than crisps made from the wild-type (4376 ng/g acrylamide in DSpco5, Figure 9D).

### 2.6. Mutations in the VInv and AS1 Do Not Significantly Affect Tuber Width and Length of Most Events

**Figure 10:**
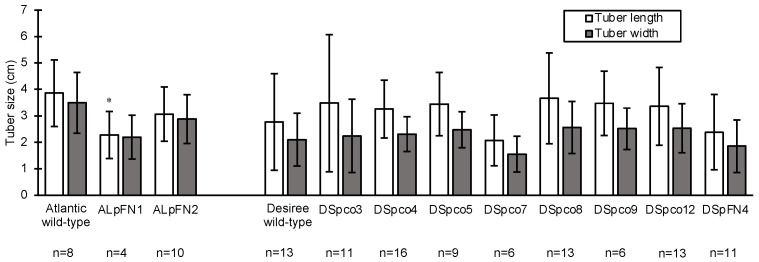
Size of tubers of wild-type and gene-edited events of Atlantic and Desiree. The bars represent the mean tuber length or width and standard deviations for replicate tubers and three technical measurements for each replicate tuber. n = the number of replicates. The asterisk (*) indicates measurements significantly (*p* < 0.05) different from those of the wild-type.

The edited events and wild-type plants were grown in pots in a glasshouse for two months before harvesting. The width and length of tubers were compared to assess if mutation of the target genes affected the size of tubers. The tuber length (stem to distal ends) of the Atlantic events ranged from 1.5 cm to 5 cm at harvest, and those of Desiree events were between 0.7 cm to 6 cm long (Figure 10). The mean tuber length of replicates of the ALpFN2 event was comparable to that of wild-type (*p* < 0.05), but that of event ALpFN1 was significantly shorter (*p* < 0.05, Figure 10). The mean tuber width of the two Atlantic events and the mean length and width of tubers of the Desiree events were not significantly different from the wild-type (*p* < 0.05, Figure 10). Thus, overall, there was no significant impact of mutations in the *VInv* and *AS1* genes on the size or shape of the tubers except for the tuber length of ALpFN1.

## 3. Discussion

We successfully used the CRISPR-Cas9 system and multiple sgRNAs to edit the *VInv* and *AS1* genes in both Atlantic and Desiree potato cultivars, generating events with mutations in either one or both genes. Cold-stored tubers of some of these events had reduced glucose and fructose concentrations. Crisps processed from them were lighter in colour with lower acrylamide concentrations. The significant reduction in browning and acrylamide concentrations in the crisps made from the two Atlantic events is noteworthy, as it demonstrates that CRISPR-Cas9 gene-editing could be used to improve one of the most popular potato chipping varieties, which has a tendency to accumulate reducing sugars [12]. The acrylamide concentrations in the two events (365.4 ng/g in ALpFN1 and 332.9 ng/g in ALpFN2) were below the only global benchmark for acrylamide in food set by the European Commission Regulation, which is 500 µg/kg (500 ng/g) acrylamide for French fries and 750 µg/kg (750 ng/g) acrylamide for potato crisps or other potato products [38], further providing evidence that CRISPR-based editing has great potential as a new tool for potato improvement. This is especially important because although potatoes with low browning and acrylamide-forming potential are preferred by the industry for healthy fries and crisps, such cultivars are not readily available. This is because the potato is an outcrossing polyploid species, and cultivars have a high level of heterozygosity, making the breeding and selection of new traits complex and a prolonged process [39]. 

For the CRISPR-Cas9 system to be effective as a breeding tool for rapid generation of new or improved potato varieties with desirable traits, it must be possible to modify simultaneously all alleles of a gene or, in the case of a polygenic trait, of more than one gene. To achieve this, it may be necessary to use multiple sgRNAs to achieve relatively high mutation frequencies [40,41]. Our study demonstrated that this is possible: in our research, we obtained mutations in both target genes, *VInv* and *AS1*, in 58% and 100% of generated Desiree and Atlantic transgenic events, respectively. Similarly, Zhao et al. [33] simultaneously edited the *Sbe1* and *Sbe2* genes of the potato cultivar Desiree with multiple sgRNAs. This resulted in 72% of regenerated lines carrying at least one mutated allele compared to 52% when a single gene was targeted. However, when the *StGBSS1* and *StDMR6*-1 genes were targeted for editing with multiple sgRNAs in the potato cultivar Desiree in another study, only the *StDMR6-1* was edited [36]. The differences in the results suggest that the success of multiplex gene editing may depend on many factors, including the efficiency of the specific sgRNAs used. Multiplexing sgRNAs can be expected to increase the speed and efficiency with which new phenotypes can be developed, and it will be particularly valuable for improving polygenic traits in species such as potatoes [33,40]. 

Biotechnology-based methods to reduce browning and acrylamide formation in high-temperature-processed potato products made from cold-stored tubers have focused on lowering the accumulation of fructose, glucose, and amino acids, particularly asparagine, with varying success. These aims have been achieved to some extent via RNA interference of the *VInv* and/or *AS1* and *AS2* genes or via overexpression of invertase inhibitors in some cultivars such as Atlantic, Katahdin, Russet Burbank, Karaka and Ranger Russet [14,16,18,22,42,43]. Inhibition of the activities of VINV or AS1 alone can reduce the acrylamide-forming potential to some extent in crisps of some potato cultivars [14,15]. By using the CRISPR-Cas9 system to edit the *VInv* and *AS1* at the same time, we achieved a significant reduction (up to 80%) in acrylamide concentration in seven of the eight events with both genes edited and up to 86% in DSpco12, a single *VInv*-edited event. The result is in line with Zhu et al. [18], who achieved up to 93% acrylamide reduction in crisps made from Russet Burbank using RNAi to silence the *VInv*, *AS1* and *AS2* genes. Reducing the activity of the VINV with or without a corresponding reduction in the expression of the AS1 and/or AS2 has been shown to result in lighter-coloured crisps and reduced acrylamide-forming potential in some cultivars, indicating that genotypic characteristics of potato lines and cultivars may play a role in the effectiveness of any new breeding technology to improve a cultivar. For example, for any potato cultivar, the role of the three genes in browning and acrylamide-forming potential may have to be established so the one or those with the most critical roles could be targeted. There is a lot to gain in targeting and silencing specific genes that may play vital roles in the expression of traits, as this will reduce cost, effort and potential off-target silencing that may affect other desirable agronomic traits. 

It might be expected that a reduction in glucose and fructose concentrations would be balanced by an increased sucrose accumulation in the cold-stored tubers of the edited events, as less sucrose would be hydrolysed because of the disruption to VINV activity. However, this was not the case, except for the event ALpFN1. Reduced VINV activity may not have changed the sucrose concentration substantially in the events, or other enzymes, such as sucrose synthase, may have broken down the sucrose. Sucrose synthase is the predominant enzyme that breaks down sucrose in sink tissues, but when tubers are detached from the plant and stored, its activity declines, and VINV activity becomes predominant [17,44,45]. It is possible that in the events with reduced activity of VINV, other metabolic activities, requirements of the tubers or the re-activation of sucrose synthase induced the hydrolysis of accumulated sucrose. These results suggest that the browning of high-temperature processed potato products may be influenced by factors additional to the level of sucrose in cold-stored tubers. 

Our results and many others demonstrate that potato cultivars can be modified with beneficial agronomic traits with health benefits using new breeding technologies, such as the CRISPR-Cas system, RNAi and over-expression of endogenous and exogenous genes [46,47,48]. Most of these techniques involve introducing and integrating foreign DNA in the form of DNA vectors, which contain genes, promoters and selectable marker genes derived from other species, into the genome of plants, making the final products transgenic or GM. Although developing GM crops is much less laborious and more precise than conventional breeding methods, their commercialization is fraught with difficulties, problems or risks, depending on who makes the judgement. It currently costs more and takes longer to bring these products to market because of regulatory hurdles in most countries [49]. Moreover, more time and effort are needed to educate consumers on the stigma of potential health risks associated with GM products. In most cases, consumer hesitancy in the consumption of GM products is borne out of fear of the unknown and a lack of education on the scientific processes involved in genetic manipulations. Despite these constraints, over 10% of the world’s arable land is cultivated to GM crops, and the increasing marketing of these crops around the globe indicates the negative consumer sentiments towards GM crops are waning, and the crops’ due contribution to the push for global food security is becoming a reality [50]. Among these are the 51 GM potato cultivars currently recorded on the International Services for the Acquisition of Agri-Tech Applications (ISAAA) databases as commercial GM potatoes. They include the commercialized Innate^®^ potato, which has stacked RNAi traits, including lowered free asparagine and reducing sugars. These examples strengthen the need for GM potato cultivars such as the ones we generated in this study. 

In jurisdictions where GM potatoes with improved traits are not marketable, non-GM potatoes with equivalent agronomic traits, such as those that can be generated with the CRISPR-Cas system using external RNPs, may be valuable alternatives. RNP-particle bombardment is a relatively new strategy for gene-editing plants. It has been applied once in a CRISPR study on potatoes, but no mutation frequency was reported [51]. In our study, the mutation frequency from RNP-particle bombardment was 0.44%, comparable to the 0.56% reported for wheat [52]. The primary reason for the lower mutation frequencies for RNP-particle bombardment is that the RNPs are active or functional for a relatively shorter period after introduction into cells or tissues. In contrast, for transgenic plants, DNA vectors encoding the Cas9 and sgRNA are integrated into the potato genome, thereby increasing the chances of continuous production, complexing and activity of RNPs in transgenic cells. The RNP-bombarded cells are, therefore, less-exposed to the editing machinery than transgenic cells, which also undergo selection pressure. Despite the low frequency of editing, the RNP-particle bombardment approach holds real potential for several reasons. It generates non-transgenic events with site-directed nuclease 1 (SDN-1) editing, an outcome of gene editing, which results in a deletion, insertion, or substitution of a base or bases or a combination of the mutations without the introduction of foreign DNA for repair via endogenous non-homologous end-joining. SDN-1 food crops or the process of SDN-1 editing are currently not regulated as GMOs in many jurisdictions (e.g., USA, Canada, Argentina, Japan, and Australia) or are in the process of being deregulated in many other countries, where gene-edited plants and food products will likely make a vital contribution to food security [53,54,55,56]. Notably, the RNP approach is a relatively cheaper and quicker process of generating new varieties of plants, as it avoids the generation of plant transformation vectors and the associated cumbersome selection processes during the tissue culture of explants. The process does not introduce transgenes into existing cultivars, avoiding some regulatory and financial limitations of developing and bringing transgenic crops to market. Thus, the advantages of using the RNP approach to generate potato cultivars with new or improved agronomic traits include speeding up breeding and commercialization processes, as it avoids the complexities associated with conventional breeding, the potential loss of existing desirable traits in a cultivar and the regulatory and financial barriers associated with GM crops. 

With the generation of the SDN-1 events in this study, the Research Stage of the Commercialization Pathway, the journey to bringing laboratory research to the market, which includes the “Gene/Trait Discovery” phase and the proof-of-concept phase of the “Development Stage”, has been achieved successfully [57]. The other phases of the Development and Commercialization stages will slightly differ for the RNP-generated and GM-edited events. To advance these events to commercialization, it would be of interest to characterize them further to understand the main factor(s) behind the improvement and to distinguish possible chimeras among the events by studying those which will carry the mutations to subsequent generations. For the GM-edited events, obtaining multiple clones will facilitate field trials to obtain data to ensure critical performance indicators such as yield and tolerance to stresses are not affected by the editing. For the RNP approach, the DSRNP217 development was behind the GM events because of the time they were generated. We could not synchronise this event’s clones with those of the GM-edited events in time for the characterization experiments because of COVID-19 restrictions in Western Australia. The first step towards commercialization is to obtain clones and characterize them in detail. Further phenotypic and genotypic characterization is being explored to develop the event for field assessment and market viability. Increasing the efficiency of the RNP-particle bombardment to generate more events with different forms of editing of one or both target genes will dramatically impact the output and commercialization of the event. This could be conducted by optimizing the ratios of Cas9 and gRNA in the RNP complex to deliver the best mutation rates, increasing the penetration of the RNPs into cells by increasing the number of bombardments, or by using cell-penetrating peptides or improving the mutation efficiencies by using protoplasts as explants, as RNPs have been demonstrated to increase mutation rates in some potato cultivars by up to 68% when used to edit genes in protoplasts [58,59]. This aspect is worth the attention, as non-GM crops are currently not regulated by gene technology regulators and are accepted by well-informed consumers in many countries. An example is the recent CRISPR-edited wheat with silenced asparagine synthetase gene *TaASN2*, which is now being assessed in field trials in the UK [60,61]. This trial and the recent announcement of a CRISPR gene-edited tomato for commercial sale in Japan represent a significant advance for the commercialization of gene-edited crops. Hopefully, potato cultivars with low potential for acrylamide formation and CIS will not be far behind.

## 4. Materials and Methods

### 4.1. Amplification and Sequencing of Portions of the Vlnv and AS1 Genes Used for sgRNA Design

Preliminary assessment of the reference sequences of the *VInv and* AS1 genes at the NCBI databases established the presence of PAM sites appropriate for the design of sgRNAs in the first five exons of both genes. Based on this information, the coding sequence of the *VInv* gene (GenBank accession no. HQ110080.1) was used to design the primer pair VInv1-F (5′-CACTGGCTCTACTTGCCTTT-3′) and VInv1-R (5′-GGGTTATCGGGTGTCCATTTAT-3′), which amplified a fragment of the third exon of the gene (593 bp). For the *AS1* gene (GenBank accession no. XM_006343993.2), the primer pair AS1-F (5′-GGCTTTGTTGGGTTGTTCGG-3′) and AS1-R (5′-TAGAGTACAAGTGCCCCGGA-3′) was used to amplify a 530 bp (exons 1 to 5) fragment from cDNA because of the presence of long introns in the selected region of the gene. The fragments were amplified from potato cultivars Atlantic and Desiree, edited in this study. For each cultivar, 100 mg of leaves of in vitro-cultured plants were used for DNA and RNA extraction using the CTAB and TRIzol (Invitrogen, Waltham, MA, USA, catalogue no. 15596026) methods, respectively [62,63]. The GoScript™ Reverse Transcriptase (Promega Corporation, Madison, WI, USA, catalogue no. PAA5000) was used to synthesise cDNA from 80 ng of RNA according to the manufacturer’s protocol. The PCRs were conducted with the GoTaq^®^ Green Master Mix (Promega Corporation, catalogue no. M7122) and used 300 ng of cDNA or 100 ng of DNA for each potato cultivar in separate 20 µL reactions. The PCRs were set up in an Applied Biosystems Veriti Thermal Cycler (Thermo Fisher Scientific, Waltham, MA, USA) with the following profile: initial denaturation at 95 °C for 5 min followed by 30 cycles of 95 °C for 30 s, 55 °C for 30 s, 72 °C for 40 s; and with a final extension at 72 °C for 7 min.

The amplicons were analysed on a 1% agarose gel (Fisher Biotec, Wembley, Australia, catalogue no. AG100) and purified using the Wizard^®^ SV Gel and PCR Clean-Up System (Promega Corporation, catalogue no. A9281). They were ligated to the pGEM-T Easy vector using an insert-to-vector molar ratio of 3:1 and multiplied using *Escherichia coli* JM109 competent cells (Promega Corporation, catalogue no. A3600 and L2005), following the manufacturer’s protocols. Five (out of 20) microlitres of a suspension of ampicillin-selected white bacterial colonies were further screened by PCR to confirm they contained ligated plasmid DNA using the conditions described above. The Universal M13 primers (M13-F: 5′-GTAAAACGACGGCCAGT-3′ and M13-R: 5′-CAGGAAACAGCTATGAC-3′) were used for the PCR and sequencing of plasmid DNA isolated from overnight cultures of selected bacterial colonies. The selected bacteria colonies were cultured using the media and conditions recommended for the *Escherichia coli* JM109 competent cells (Promega Corporation). The plasmid DNA was sequenced using the BigDye^®^ Terminator v3.1 Chemistry, the recommended PCR conditions and the ethanol/EDTA precipitation method for the PCR products described in the BigDye^®^ Terminator v3.1 Cycle Sequencing Kit protocol (Applied Biosystems, Waltham, MA, USA). The chromatograms were analysed using the Geneious Prime software (Biomatters Ltd., San Diego, CA, USA). 

### 4.2. Design and Testing of the Efficiency of sgRNAs Targeting the Vlnv and AS1 Genes

Sequences of different clones of amplicons of the *Vlnv* and *AS1* genes were aligned with the reference sequences for *VInv* (accession no. HQ110080.1) and *AS1* (accession no. XM_006343993.2) using the Multalin web tool with standard settings [64]. Conserved regions without allelic polymorphism were used to select sgRNA target sites using the Cas-Designer [65]. Two 20-nt target sequences were selected for each gene; g67 and g10 for the *Vlnv* gene and g4 and g7 for the *AS1* gene. 

To assess the efficiency of the sgRNAs in vitro in Cas9 cleavage assay, each sgRNA with gRNA scaffolds in tandem was first transcribed in vitro from DNA templates. The templates were constructed using overlapping PCRs and the pUC119-gRNA vector (Addgene plasmid #52255; RRID:Addgene_52255; http://n2t.net/addgene:52255; accessed on 5 June 2019) as described by [66]. For each sgRNA, the final DNA construct consisted of a T7 promoter sequence, the single guide sequence and the gRNA scaffold. The details of the primers, the overlapping PCR strategy and the PCR conditions were as described previously [66,67]. The DNA construct for each sgRNA was transcribed in 20 µL reactions using the HiScribe™ Quick T7 High Yield RNA Synthesis Kit (New England Biolabs, NEB, Ipswich, MA, USA, catalogue no. E2050S). Each reaction consisted of 0.75 X reaction buffer, 7.5 mM each of ATP, GTP, CTP, UTP, 1.5 μL of T7 RNA polymerase mix and 1 μg of template DNA. They were incubated at 37 °C for 14 hr in a thermal cycler. The transcripts were purified using the chloroform extraction and sodium acetate-ethanol precipitation protocol recommended for RNA synthesised with the HiScribe kit. Before the purification, template DNA was removed from the transcripts using TURBO DNase I (Thermo Fisher Scientific, catalogue no. AM2238) following the manufacturer’s protocol. 

Cas9 cleavage assays were conducted on amplicons (593 bp and 530 bp, respectively, for the *VInv* and *AS1*) generated from the potato cultivars Atlantic and Desiree. The 20 µL reactions consisted of a 10:10:1 molar ratio of Cas9: gRNA: target amplicon. First, a 10 µL reaction containing 0.01 nM of Cas9 (Proteowa Pty Ltd., Murdoch, Australia), 0.01 nM of gRNA and 1X NEBuffer 3.1 (New England Biolabs, NEB, Ipswich, MA, USA, catalogue no. B7203) was incubated at room temperature for 10 min, then 10 µL of 0.001 nM target amplicon was added, and the NEBuffer 3.1 was topped up to 1X final concentration. The reactions were incubated at 37 °C for 15 min and then analysed via electrophoresis using a 2% agarose gel.

### 4.3. Construction of Gene-Editing Vectors 

The two Cas9-encoding gene-editing vectors, pFGC-pcoCas9-ASVI and pFN117-Cas9-ASVI, used for transformation, were generated based on the vectors pFGC-pcoCas9 (Addgene plasmid # 52256) and pFN117-Cas9 (donated by Dr. Fatima Naim, Queensland University of Technology), each of which was modified with an ASVI guide RNA cassette. The pFGC-pcoCas9 and pFN117-Cas9 vectors have different regulatory translational and transcriptional elements for the Cas9 sequence, as shown in the vector maps in Supplementary Figure S1. The ASVI cassette was designed with the *Arabidopsis thaliana* U6 promoter (AtU6-1) sequence driving the four sgRNAs with gRNA scaffolds in tandem, each separated by a sequence of the endogenous plant transfer RNA (tRNA) and ending with the Pol III terminator sequence. The *XhoI* and *EcoRI* restriction sites were added upstream of the AtU6-1 promoter, and the *PacI* and *KpnI* restriction sites were added downstream of the terminator sequence. The cassette was synthesised commercially (GENEWIZ/Azenta, Burlington, MA, USA) and ligated to pFGC-pcoCas9 using the *EcoRI* and *PacI* restriction sites to create vector pFGC-pcoCas9-ASVI and to the pFN117-Cas9 using the *XhoI* and *KpnI* restriction sites to generate vector pFN117-Cas9-ASVI (Supplementary Figure S1). The ligations were conducted with a 3:1 insert: vector molar ratio using the ligase in the pGEM^®^-T Vector System, the plasmid DNA multiplied with the *E. coli* JM109 competent cells and purified using the Wizard^®^ Plus Minipreps DNA Purification System using the recommended protocols provided by Promega Corporation and described in detail above. 

The vectors were sequenced to confirm their integrity using the BigDye^®^ Terminator v3.1 Chemistry, the reactions were cleaned up, and the raw sequence files were edited using the procedures described above. The primers for sequencing were the FGC-F (5′-GAAATTCAGGCCCGGTTGCC-3′) and FGC-R (5′-CTAGGATAAATTATCGCGCGCGGTG-3′) for vector pFGC-pcoCas9-ASVI and, FN117-F (5′-CATAACGTGACTCCCTTAATTCTCC-3′) and FN117-R (5′-CATGTTGACCTCCAAGCTTGAATTC-3′) for vector pFN117-Cas9-ASVI (Supplementary Figure S1). Competent *A. tumefaciens* strain GV3101 cells were modified with the vectors using the freeze–thaw method described by [67]. 

### 4.4. Maintenance of Plants and Agrobacterium-Mediated Transformation

Plantlets of both Atlantic and Desiree, from which leaves were harvested for transformation, were maintained in vitro through regular subculturing of nodal segments on Murashige and Skoog (MS) medium (Sigma, St. Louis, MO, USA, catalogue no. M5519) supplemented with 20 g/L sucrose (pH 5.6) and solidified with 2.8 g/L Gelrite (Sigma-Aldrich, St. Louis, MO, USA, catalogue no G1910). To obtain broader leaves for transformation, two-week-old in vitro plantlets were transferred to a potting mix (40 kg of soil mixed with 20 g of dolomite, 15 g of CaCO_3_, 40 g of Grower’s blue, 40 g of Osmocote^®^ all-purpose fertiliser) and maintained in a growth chamber at 30 °C, under a 16 h light/ 8 h dark photoperiod for at least four weeks. Fully expanded leaves were harvested, washed under a running tap for 15 min and then surface sterilised by dipping in 70% ethanol for 30 s, followed by incubation for 40 s in 1% sodium hypochlorite solution containing three drops of Tween-20. The leaves were washed four times with sterile water and soaked for two minutes in fresh sterile water with occasional swirling. Leaf discs were prepared by punching the sterile leaves with a sterile cork-borer (0.7 cm in diameter); they were kept in liquid MS medium (pH 5.6) until transformed with modified *A. tumefaciens* cultures.

The leaf disc transformation was carried out as described by [68] with some modifications. Overnight bacterial culture (OD_600_ = 0.8) was pelleted at 5000× *g* for 20 min and was resuspended in liquid MS medium supplemented with 30 g/L sucrose, and 100 µM acetosyringone. The inoculation procedure involved immersing 30 leaf discs in bacterial suspension in a 90 mm Petri dish for 30 min with gentle shaking by placing the dish on a rocking shaker at 30 rpm. After inoculation, the leaf discs were transferred to a callus induction medium (CIM: MS media with Gamborg’s B5 vitamins [MSB5, PhytoTech Labs, catalogue no. M404] + 5 mg/L NAA, 2 mg/L BAP, 16 g/L glucose [pH 5.6], 2.8 g/L Gelrite) and maintained for two days in the dark. Afterwards, the discs were washed once with 200 mg/L Timentin and transferred to CIM supplemented with 200 mg/L Timentin. Five days later, Desiree leaf discs were transferred onto shoot induction medium (SIM) 1 (MSB5 + 0.02 mg/L NAA, 0.15 mg/L GA_3_, 2.2 mg/L zeatin riboside, 16 g/L glucose [pH 5.6] 2.8 g/L Gelrite, 200 mg/L Timentin), whereas the Atlantic leaf discs were transferred to SIM 2 (MSB5 + 6 mg/L BAP, 5 mg/L GA_3_, 16 g/L glucose [pH 5.6], 2.8 g/L Gelrite, 200 mg/L Timentin). Shoots that developed were individually transferred to a rooting medium made with MS, 20 g/L sucrose and 2.8 g/L Gelrite (pH 5.6). The shooting and rooting media had 0.5 mg/L glufosinate-ammonium for selecting events generated with pFGC-pcoCas9-ASVI or 50 mg/L kanamycin for selecting events generated with pFN117-Cas9-ASVI.

### 4.5. RNP-Particle Bombardment

Gold particles (0.6 μm in diameter) used for bombardment were freshly prepared as described for the Biolistic^®^ PDS-1000/He Particle Delivery System (Bio-Rad Lab. Inc., Berkeley, CA, USA) with some modifications. After sterilisation with 70% ethanol, the particles were resuspended in 5 μL of NEB 3.1 buffer and 45 μL of nuclease-free water at a final concentration of 90 mg/mL. The RNP complex for each sgRNA was assembled separately in 25 µL reactions comprising 5 µg of *in vitro*-transcribed gRNA-scaffold, 5 µg of Cas9 protein (Proteowa Pty Ltd.) and 1X NEB 3.1 buffer in nuclease-free water. The reactions were incubated for 15 min at 37 °C. For the bombardment, 25 µL of each RNP complex was added to 50 µL of the sterilised gold particles, mixed gently and 15 μL spread on a microcarrier and air-dried in a laminar flow hood before bombardment. Leaf discs to be treated with the RNPs were previously cultured on CIM for five days. Two to four hours before treatment, the discs were transferred onto Petri dishes containing MS solid medium supplemented with 20 g/L sucrose, 0.2 M mannitol (pH 5.6), and 2.8 g/L Gelrite. The discs were bombarded twice with freshly prepared RNP-coated particles at a 9-cm target distance with the PDS-1000/He™ Biolistic system (BioRad Lab. Inc.) using 1100 psi rupture discs. Bombarded leaf discs were transferred and maintained on CIM for two days before transfer to SIM 1 (Desiree) or SIM 2 (Atlantic). Developing shoots were transferred to the rooting medium described in Section 4.4. 

### 4.6. Screening of Gene-Edited Plants 

The transgenic status of regenerated events was assessed using PCRs to establish the integration of Cas9 DNA into the genome by amplifying a portion of the coding sequence. For plants transformed with the pFGC-pcoCas9-ASVI, the primers PFGC9-IF (5′-GAGGAAACTCTCGTTTCGCTTGG-3′) and PFGC9-IR (5′ GCTTTGGAAGAACCTTCTCGT-3′) amplified a 351 bp of the Cas9 DNA, whereas the primers PFN117C9-F (5′-GACGGCACCGAGGAACTG-3′) and PFN117C9-R (5′ TCGTTGGGCAGGTTCTTATC-3′) amplified the 365 bp of the Cas9 coding sequence in the vector pFN117-Cas9-ASVI. In addition, a fragment of the T-DNA integrated into the generated events was amplified to confirm the transgenic status of each event further. For the events transformed with pFGC-pcoCas9-ASVI, the primers BAR-F (5′-CACGGTCAACTTCCGTACC-3′) and BAR-R (5′- CAGATAAAGCCACGCACATTTAGG-3′) amplified a 654 bp fragment made up of part of the *BlpR* gene and the MAS terminator sequence. Events transformed with vector pFN117-Cas9-ASVI were also screened with primers FN117-F (5′-CATAACGTGACTCCCTTAATTCTCC-3′) and FN117-R (5′-CATGTTGACCTCCAAGCTTGAATTC-3′), which together amplified 1120 bp of the gRNA cassette. 

### 4.7. Detection of Mutation in Gene-Edited Plants by PCR and Sequencing 

Mutations in the RNP-treated and transgenic plants were determined by sequencing amplicons of the *VInv* and *AS1* genes obtained with the primer pairs 67-F (5′-GGGGAAATATCACATGGGGC-3′) and 10-R (5′-AGTGCAATACCCGTTTTACCAA-3′) for the region containing g67 and g10 target sites; 4-F (5′-GGCTTTGTTGGGTTGTTCGG-3′) and 4-R (5′-AACCCTTCAATGCACAGACAG-3′) for the region containing g4 target site and 7-F (5′-CCTAACGTGGGATAAGAAATCTCT-3′) and 7-R (5′-GATGTGCCAAGTAAAAATCACCA-3′) for the region including the g7 target site. PCRs, done with 100 ng of genomic DNA isolated from 100 mg of leaf tissues following the CTAB method, were conducted using the GoTaq^®^ Green Master Mix with the temperature profile described under Section 4.1. The amplicons were sequenced directly and, where required, were cloned using the pGEM-T Easy vector system as described above. Between 18 and 35 clones of each amplicon were sequenced to identify mutation type and frequency. 

### 4.8. Planting, Maintenance of Edited Plants in Soil and Harvesting of Tubers

To analyse the effect of editing on the growth and development of the events and to obtain tubers for further characterisation, the events with wild-type Atlantic and Desiree were cloned through subculturing of nodal segments on MS medium (Sigma-Aldrich, St. Louis, MO, USA, catalogue no. M5519) supplemented with 20 g/L sucrose (pH 5.6) and solidified with 2.8 g/L Gelrite (Sigma-Aldrich, St. Louis, MO, USA, catalogue no G1910). Three clones for each of the ten gene-edited events and wild-type were transferred from the rooting media (described above) to pasteurised potting mix (5 L pot for each plant), and the plants were maintained in a Physical Containment Level 2 (PC2) glasshouse at Murdoch University, Western Australia from August to November 2020. The tubers were harvested, washed and air-dried. Tuber width and length were measured with a ruler, after which they were placed in paper bags and stored at 4 °C in the dark for four months before being subjected to sugar content analyses and high-temperature processing. 

### 4.9. Estimation of Sucrose, Glucose, and Fructose Concentrations in Cold-Stored Tubers

Total soluble sugars were extracted from each cold-stored tuber separately using 80% (*v*/*v*) ethanol, as described previously by McKibbin et al. [69]. Sugar quantification assay was conducted according to the method of Jones et al. [70] with modifications made for assessment in a plate reader, as described by McKibbin et al. [69]. Briefly, soluble sugars were extracted from 300 mg of homogenised tuber tissue by adding 1 mL of 80% ethanol, followed by incubation at 70 °C for an hour with continuous agitation. The supernatant was collected by centrifugation at 10,000× *g* for 2 min. The assay was performed in a microtiter plate; each 200 µL reaction contained 100 mM imidazole, 10 mM MgCl_2_, 1.1 mM ATP, 0.5 mM NADP, 0.14 U glucose-6-phosphate dehydrogenase (Sigma-Aldrich, St. Louis, MO, USA, catalogue no. G6378) and 20 µL of the tuber extract (previously cooled to room temperature). Absorbance was measured using a Beckman Coulter DTX 880 Multimode Detector microplate reader with an A340 nm absorbance filter and 10 nm bandwidth. Initial absorbance was recorded as the baseline for each reaction. For glucose, fructose and sucrose quantification, 0.12 U hexokinase (Sigma-Aldrich, St. Louis, MO, USA, catalogue no. H4502), 1 U phosphoglucose isomerase (Sigma-Aldrich, St. Louis, MO, USA, catalogue no. P5381) and 4 U invertase (Sigma-Aldrich, St. Louis, MO, USA, catalogue no. I4504) were added accordingly; each was followed by a 10-min incubation at room temperature, and absorbance was recorded. Three readings were recorded for each sample after adding the enzyme. Standard curves were generated for each sugar type using concentrations of 0 to 100 nmol, and sugar concentration in each sample was determined by plotting the absorbance values against the standard curves.

### 4.10. High-Temperature Processing of Cold-Stored Tubers into Crisps and Acrylamide Assay

The cold-stored tubers of the edited events and wild-type were peeled, precisely cut into 2 mm thick slices using a mandoline slicer and deep-fried in vegetable oil at 191 °C for 60 s. Because there were more events of Desiree, the slices were fried in batches, with each batch including a wild-type. The colour intensity of the crisps was quantified using the FIJI image processing software, which assigns a value of 255 to the brightest (white) colour and zero to the darkest (black) colour [37]. The acrylamide concentration in the crisps was estimated from 2 g of homogenised crisps using the Acrylamide-ES ELISA kit (Eurofins, Brisbane, AUS, catalogue no. 515680) according to the manufacturer’s instructions.

### 4.11. Statistical Analyses

The Welch Two Sample t-test was used to determine significant differences in the mean values assigned to the crisp colour intensities, the means of the sucrose, fructose and glucose concentrations in the cold stored tubers, the mean concentrations of acrylamide in the crisps, and the mean tuber lengths and widths between the wild-types and gene-edited events. Statistical analysis was performed using R ver.3.6.3 [71], and statistical significance was assessed at *p* < 0.05. Bar charts were generated using Microsoft Excel.

## Figures and Tables

**Table 1:** Transgenic events created with two gene editing vectors and the status of mutations in the two target genes. A “+” indicates mutation in the gene, and N indicates no edit detected in sequence.

Transformation Vector	Potato Cultivar	Transgenic Event	Mutation at Target Site	
			*AS1* Gene	*VInv* Gene
pFN117-Cas9-ASVI	Atlantic	ALpFN1	+	+
		ALpFN2	+	+
	Desiree	DSpFN1	+	+
		DSpFN2	+	+
		DSpFN3	+	+
		DSpFN4	+	+
		DSpFN5	+	+
		DSpFN6	+	+
		DSpFN7	+	+
		DSpFN8	+	+
pFGC-pcoCas9-ASVI		DSpco1	N	N
		DSpco2	N	+
		DSpco3	+	+
		DSpco4	+	+
		DSpco5	N	+
		DSpco6	N	N
		DSpco7	+	+
		DSpco8	+	+
		DSpco9	+	+
		DSpco10	+	+
		DSpco11	N	+
		DSpco12	N	+

## Data Availability

Not applicable.

## Supplementary Materials

The following supporting information can be downloaded at: https://www.mdpi.com/article/10.3390/plants12020379/s1, Figure S1: Schematic diagram showing the map of the gRNA cassette and the creation of the binary vectors used for potato leaf disc transformation. The diagram shows a representation of the ASVI gRNA cassette (the Arabidopsis thaliana AtU6-1 promoter, each of the coding sequences of four sgRNAs and gRNA scaffolds separated by the transfer RNA (tRNA) processing sequences) and maps of vectors pFGC-pcoCas9-ASVI and pFN117-Cas9 also showing the primer pairs used for sequencing plasmids to confirm vector integrity and for confirming the transgenic status of generated events via PCRs.
