# Supplementary material for: Multiplex CRISPR-Cas9 Gene-Editing Can Deliver Potato Cultivars with Reduced Browning and Acrylamide
Source: Plants (Basel). 2023 Jan 13;12(2):379. doi: 10.3390/plants12020379 (PMC9864857; doi:10.3390/plants12020379)
Supplement: Supplementary file 1 [file plants-12-00379-s001.zip › plants-1979280-supplementary.pptx]

## Slide 1
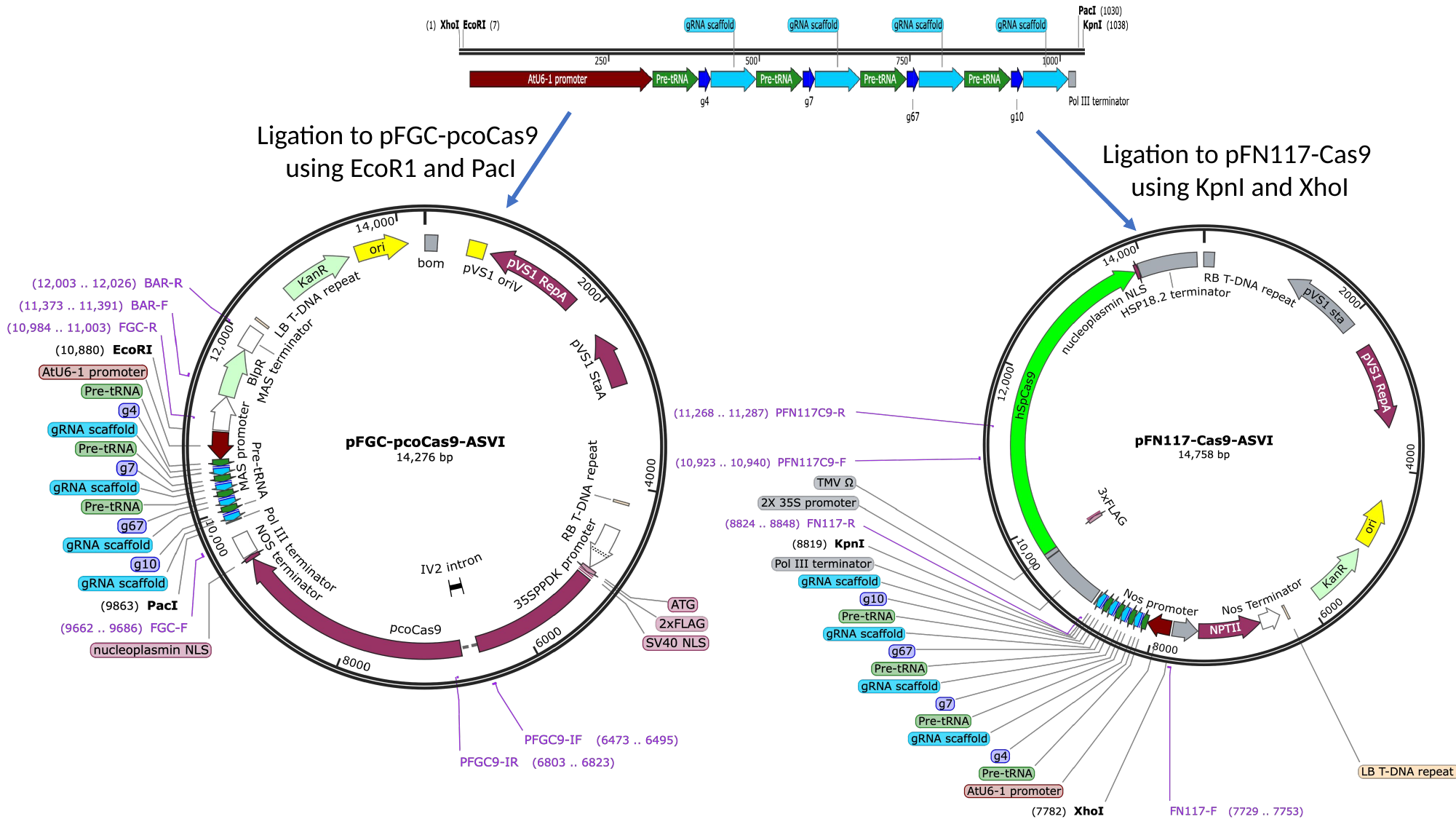

Ligation to pFGC-pcoCas9
using EcoR1 and PacI
Ligation to pFN117-Cas9
using KpnI and XhoI

## Slide 2
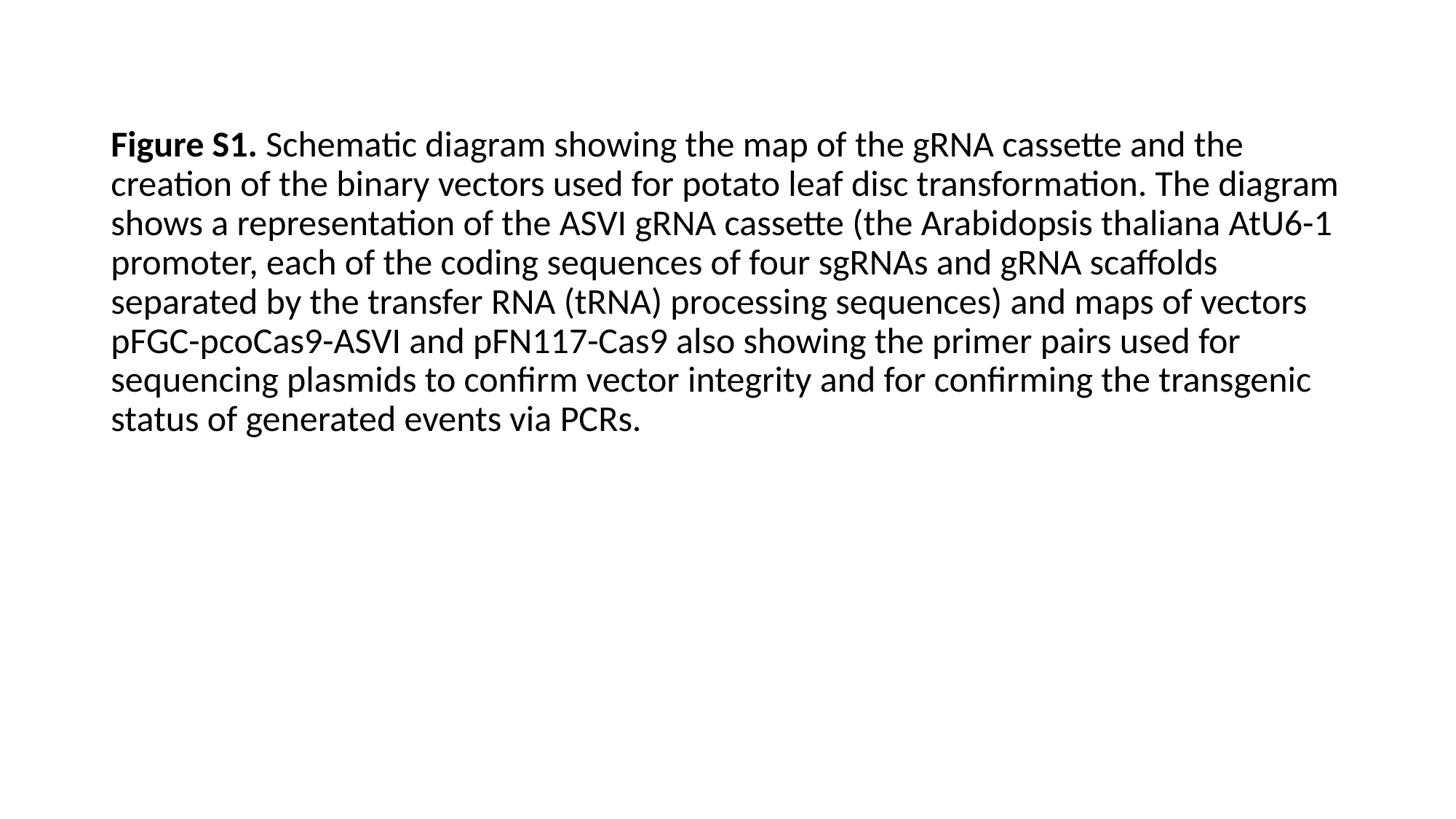

Figure S1. Schematic diagram showing the map of the gRNA cassette and the creation of the binary vectors used for potato leaf disc transformation. The diagram shows a representation of the ASVI gRNA cassette (the Arabidopsis thaliana AtU6-1 promoter, each of the coding sequences of four sgRNAs and gRNA scaffolds separated by the transfer RNA (tRNA) processing sequences) and maps of vectors pFGC-pcoCas9-ASVI and pFN117-Cas9 also showing the primer pairs used for sequencing plasmids to confirm vector integrity and for confirming the transgenic status of generated events via PCRs.
